# Supplementary material for: MetaQTL: a package of new computational methods for the meta-analysis of QTL mapping experiments
Source: BMC Bioinformatics. 2007 Feb 8;8:49. doi: 10.1186/1471-2105-8-49 (PMC1808479; doi:10.1186/1471-2105-8-49)
Supplement: Additional File 7 — MetaQTL Package : jar file and tutorial. This Zip archive contains both the MetaQTL JAR file and the files of the tutorial. [file 1471-2105-8-49-S7.zip › org.inra.metaqtl/doc/org/metaqtl/Chromosome.html]

Chromosome


|  |  |  |
| --- | --- | --- |
| **Overview** \| **Package** \| **Class** \| **Use** \| **Tree** \| **Deprecated** \| **Index** \| **Help** | |  |
| **PREV CLASS**   **NEXT CLASS** | **FRAMES**    **NO FRAMES**     **All Classes** |  |
| SUMMARY: NESTED \| FIELD \| CONSTR \| METHOD | DETAIL: FIELD \| CONSTR \| METHOD |  |


---


## org.metaqtl Class Chromosome

```
java.lang.Object
  org.metaqtl.Chromosome
```

---

``` public class Chromosome extends java.lang.Object ```

On a Chromosome there are two types of locus :

- Marker
- QTL (Quantitative Trait Loci)

inside a mapping context which is defined by both the mapping framework
(function and unit) and the population cross design used to build this
chromosome.

---

| **Field Summary** | |
| --- | --- |
| `int` | `cg`             The number of generations of the offspring population. |
| `int` | `cs`             The size of the offspring population. |
| `int` | `ct`             The type of the offspring population. |
| `java.lang.String` | `mapName`             The name of the map in which this chromosome is defined. |
| `MetaQtlResult[]` | `metaQtlAnalysis`             The results of a meta-analysis of QTL. |
| `int` | `mfc`             The mapping function for this chromosome |
| `Qtl[]` | `mqtls`             The meta Qtls on this chromosome. |
| `double[]` | `mr`             The adjacent recombination rate between ordered markers |
| `java.lang.String[]` | `mrkNames`             The ordered marker names |
| `int` | `mut`             The mapping unit for this chromosome |
| `java.lang.String` | `name`             The name of the chromosome. |
| `int` | `nm`             The number of markers. |
| `double[][]` | `proba`             Probabilities defined along the chromosome. |
| `Qtl[]` | `qtls`             The QTLs located on this chromosome. |
| `boolean` | `skeleton`             The skeleton status for this member |
| `double` | `totd`             The total distance of the chromosome. |


**Constructor Summary**

`Chromosome()`


| **Method Summary** | |
| --- | --- |
| `boolean` | `areMarkersAroundQTL(int mrkIdxL, int mrkIdxR, int qtlIdx)`             Returns true if the QTL position is inside the marker interval which markers indices are `mrkIdxL` and `mrkIdxR`. |
| `void` | `attachQTL(IBioLocus[] qtls)`             Add |
| `void` | `attachQtl(Qtl[] qtls)` |
| `void` | `computeQtlSD(int mode)`             According to the given standard deviation computation mode this method computes for each QTL the value of the standard deviation of the QTL position. |
| `double` | `getDistance(int i)`             This methods returns the distance between the first marker and the marker with the given indice using the default mapping context. |
| `double` | `getDistance(int mrkIdx, int mapping_function, int mapping_unit)`             This methods returns the distance between the first marker and the marker with the given indice using the given mapping context. |
| `double` | `getDistanceBetween(int mrkIdxL, int mrkIdxR)` |
| `double` | `getDistanceBetween(int mrkIdxL, int mrkIdxR, int mapping_function, int mapping_unit)`             This methods returns the distance between the markers with the given indices assuming that `mrkIdxL < mrkIdxR`. |
| `static double[]` | `getDistHeterogeneity(int ncm, int[][] mrkIdx, Chromosome chrom, Chromosome ref)` |
| `static int` | `getFirstCommonMrkIdx(Chromosome chrom, Chromosome chrom2)` |
| `java.lang.String` | `getMapName()` |
| `int` | `getMarkerIdxWithName(java.lang.String string)`             This method looks if it exists a marker on the chromosome with the given name and returns its indice. |
| `java.lang.String` | `getMarkerName(int i)`             Returns the names of the marker at the i^th position on the chromosome. |
| `Qtl[]` | `getMetaQtls()` |
| `java.lang.String` | `getName()`             Returns the name of the chromosome. |
| `double[][]` | `getProba()` |
| `double` | `getQTLInMrkIntProb(int mrkIdxL, int mrkIdxR, int qtlIdx)`             This methods returns the probability that the QTL be inside the marker interval with the given right and left marker indices and the given QTL indice. |
| `double` | `getQTLInMrkIntProb(int mrkIdxL, int mrkIdxR, Qtl qtl)`             This methods returns the probability that the QTL be inside the marker interval with the given right and left marker indices and the given QTL. |
| `int` | `getQtlNumber()` |
| `Qtl[]` | `getQtls()`             This method returns a copy of the QTL located on thic chromosome. |
| `static double[]` | `getRecHeterogeneity(int ncm, int[][] mrkIdx, Chromosome chrom, Chromosome ref)` |
| `Chromosome` | `getSubChromosome(java.lang.String[] markerNames)` |
| `boolean` | `hasQTL()`             This method tests if the chromosome have a set of QTL defined. |
| `void` | `r2R()` |
| `void` | `R2r()` |
| `void` | `r2R(int type, int gen)`             Re-scale the chromosome assuming that the distances and recombination fractions are based on the frequency of recombinant genotypes R instead of the real recombination fraction r. |
| `void` | `R2r(int type, int gen)`             Re-scale the chromosome assuming that the distances and recombination fractions are based on the frequency of recombinant genotypes R instead of the real recombination fraction r. |
| `void` | `setMetaQtls(Qtl[] metaQtl)` |
| `void` | `setProba(double[][] proba)` |
| `java.lang.String` | `toString()` |
| `void` | `updateMappingContext(int mapping_function, int mapping_unit)`             Updates the mapping context of the chromosome. |

**Methods inherited from class java.lang.Object**

`clone, equals, finalize, getClass, hashCode, notify, notifyAll, wait, wait, wait`

**Field Detail**

### nm

```
public int nm
```

:   The number of markers.

---


### cs

```
public int cs
```

:   The size of the offspring population.

---


### ct

```
public int ct
```

:   The type of the offspring population.

---


### cg

```
public int cg
```

:   The number of generations of the offspring population.

---


### mfc

```
public int mfc
```

:   The mapping function for this chromosome

---


### mut

```
public int mut
```

:   The mapping unit for this chromosome

---


### skeleton

```
public boolean skeleton
```

:   The skeleton status for this member

---


### totd

```
public double totd
```

:   The total distance of the chromosome.

---


### mr

```
public double[] mr
```

:   The adjacent recombination rate between
    ordered markers

---


### mrkNames

```
public java.lang.String[] mrkNames
```

:   The ordered marker names

---


### name

```
public java.lang.String name
```

:   The name of the chromosome.

---


### mapName

```
public java.lang.String mapName
```

:   The name of the map in which this chromosome
    is defined.

---


### qtls

```
public Qtl[] qtls
```

:   The QTLs located on this chromosome.

---


### mqtls

```
public Qtl[] mqtls
```

:   The meta Qtls on this chromosome.

---


### metaQtlAnalysis

```
public MetaQtlResult[] metaQtlAnalysis
```

:   The results of a meta-analysis of QTL.

---


### proba

```
public double[][] proba
```

:   Probabilities defined along the chromosome.


**Constructor Detail**

### Chromosome

```
public Chromosome()
```


**Method Detail**

### getQTLInMrkIntProb

```
public double getQTLInMrkIntProb(int mrkIdxL,
                                 int mrkIdxR,
                                 int qtlIdx)
```

:   This methods returns the probability that the QTL be inside
    the marker interval with the given right and left marker indices
    and the given QTL indice.
    The method assumes that `mrkIdxL < mrkIdxR`

    :   **Parameters:**: `mrkIdxL` - the left marker indice.: `mrkIdxR` - the right marker indice.: `qtlIdx` - the QTL indice. **Returns:**: the probability that the QTL be inside the interval.

---


### getQTLInMrkIntProb

```
public double getQTLInMrkIntProb(int mrkIdxL,
                                 int mrkIdxR,
                                 Qtl qtl)
```

:   This methods returns the probability that the QTL be inside
    the marker interval with the given right and left marker indices
    and the given QTL.
    The method assumes that `mrkIdxL < mrkIdxR`

    :   **Parameters:**: `mrkIdxL` - the left marker indice.: `mrkIdxR` - the right marker indice.: `qtl` - the QTL. **Returns:**: the probability that the QTL be inside the interval.

---


### areMarkersAroundQTL

```
public boolean areMarkersAroundQTL(int mrkIdxL,
                                   int mrkIdxR,
                                   int qtlIdx)
```

:   Returns true if the QTL position is inside the marker
    interval which markers indices are `mrkIdxL`
    and `mrkIdxR`.
    This assumes that `mrkIdxL < mrkIdxR`

    :   **Parameters:**: `mrkIdxL` - the left marker indice.: `mrkIdxR` - the right marker indice.: `qtlIdx` - the qtl indice. **Returns:**: true if the QTL position is inside the marker interval.

---


### hasQTL

```
public boolean hasQTL()
```

:   This method tests if the chromosome have a
    set of QTL defined.

    :   **Returns:**: true if there are QTL.

---


### toString

```
public java.lang.String toString()
```

:   **Overrides:**: `toString` in class `java.lang.Object`

---


### getMarkerIdxWithName

```
public int getMarkerIdxWithName(java.lang.String string)
```

:   This method looks if it exists a marker on the chromosome
    with the given name and returns its indice. Otherwise it
    returns -1.

    :   **Parameters:**: `string` - the name of the marker. **Returns:**: the indice of the marker if exists, -1 otherwise.

---


### getDistanceBetween

```
public double getDistanceBetween(int mrkIdxL,
                                 int mrkIdxR,
                                 int mapping_function,
                                 int mapping_unit)
```

:   This methods returns the distance between the markers with
    the given indices assuming that `mrkIdxL < mrkIdxR`.
    The distance is computed using the given mapping context.

    :   **Parameters:**: `mrkIdxL` - the left marker indice.: `mrkIdxR` - the right marker indice.: `mapping_function` - the mapping function.: `mapping_unit` - the mapping unit. **Returns:**: the distance between the two markers.

---


### getDistanceBetween

```
public double getDistanceBetween(int mrkIdxL,
                                 int mrkIdxR)
```

:   **Parameters:**: `mrkIdxL` -: `mrkIdxR` - **Returns:**

---


### getDistance

```
public double getDistance(int mrkIdx,
                          int mapping_function,
                          int mapping_unit)
```

:   This methods returns the distance between the first marker and
    the marker with the given indice using the given mapping context.

    :   **Parameters:**: `mrkIdx` - the marker indice.: `mapping_function` - the mapping function.: `mapping_unit` - the mapping unit. **Returns:**: the distance between the first marker and the given one.

---


### updateMappingContext

```
public void updateMappingContext(int mapping_function,
                                 int mapping_unit)
```

:   Updates the mapping context of the chromosome.

    :   **Parameters:**: `mapping_function` - the new mapping function.: `mapping_unit` - the new mapping unit.

---


### getDistance

```
public double getDistance(int i)
```

:   This methods returns the distance between the first marker and
    the marker with the given indice using the default mapping
    context.

    :   **Parameters:**: `i` - the indice of the marker. **Returns:**: the distance betwee the first marker and the given one.

---


### r2R

```
public void r2R()
```

---


### r2R

```
public void r2R(int type,
                int gen)
```

:   Re-scale the chromosome assuming that the distances
    and recombination fractions are based on the frequency of
    recombinant genotypes R instead of the real recombination
    fraction r.

---


### R2r

```
public void R2r()
```

---


### R2r

```
public void R2r(int type,
                int gen)
```

:   Re-scale the chromosome assuming that the distances
    and recombination fractions are based on the frequency of
    recombinant genotypes R instead of the real recombination
    fraction r.

---


### computeQtlSD

```
public void computeQtlSD(int mode)
```

:   According to the given standard deviation computation
    mode this method computes for each QTL the value of the standard
    deviation of the QTL position. If no standard deviation can be
    computed then the standard deviation is set to 0.0;

    :   **Parameters:**: `mode` - the standard deviation computation mode. **See Also:**: `IMetaQtlConstants`

---


### getMetaQtls

```
public Qtl[] getMetaQtls()
```

:   **Returns:**

---


### setMetaQtls

```
public void setMetaQtls(Qtl[] metaQtl)
```

:   **Parameters:**: `metaQtl` -

---


### getQtls

```
public Qtl[] getQtls()
```

:   This method returns a copy of the QTL located
    on thic chromosome.

    :   **Returns:**: a copy of the array of QTLlocated on this chromosome.

---


### getQtlNumber

```
public int getQtlNumber()
```

:   **Returns:**

---


### attachQtl

```
public void attachQtl(Qtl[] qtls)
```

:   **Parameters:**: `mqtls2` -

---


### attachQTL

```
public void attachQTL(IBioLocus[] qtls)
```

:   Add

    :   **Parameters:**: `loci` -

---


### getMarkerName

```
public java.lang.String getMarkerName(int i)
```

:   Returns the names of the marker at the i^th position
    on the chromosome.

    :   **Parameters:**: `i` - the position of the marker. **Returns:**: the names of the marker.

---


### getName

```
public java.lang.String getName()
```

:   Returns the name of the chromosome.

    :   **Returns:**: the name of teh chromosome.

---


### getRecHeterogeneity

```
public static double[] getRecHeterogeneity(int ncm,
                                           int[][] mrkIdx,
                                           Chromosome chrom,
                                           Chromosome ref)
```

:   **Parameters:**: `ncm` -: `mrkIdx` -: `chrom` -: `ref` - **Returns:**

---


### getDistHeterogeneity

```
public static double[] getDistHeterogeneity(int ncm,
                                            int[][] mrkIdx,
                                            Chromosome chrom,
                                            Chromosome ref)
```

---


### getProba

```
public double[][] getProba()
```

:   **Returns:**

---


### setProba

```
public void setProba(double[][] proba)
```

:   **Parameters:**: `proba` -

---


### getFirstCommonMrkIdx

```
public static int getFirstCommonMrkIdx(Chromosome chrom,
                                       Chromosome chrom2)
```

:   **Parameters:**: `chromosome` -: `chromosome2` - **Returns:**

---


### getSubChromosome

```
public Chromosome getSubChromosome(java.lang.String[] markerNames)
```

:   **Parameters:**: `strings` - **Returns:**

---


### getMapName

```
public java.lang.String getMapName()
```

:   **Returns:**


---


|  |  |  |
| --- | --- | --- |
| **Overview** \| **Package** \| **Class** \| **Use** \| **Tree** \| **Deprecated** \| **Index** \| **Help** | |  |
| **PREV CLASS**   **NEXT CLASS** | **FRAMES**    **NO FRAMES**     **All Classes** |  |
| SUMMARY: NESTED \| FIELD \| CONSTR \| METHOD | DETAIL: FIELD \| CONSTR \| METHOD |  |


---
